# Genetic characterization in symptomatic female DMD carriers: lack of relationship between X-inactivation, transcriptional DMD allele balancing and phenotype

**DOI:** 10.1186/1471-2350-13-73

**Published:** 2012-08-16

**Authors:** Simona Brioschi, Francesca Gualandi, Chiara Scotton, Annarita Armaroli, Matteo Bovolenta, Maria S Falzarano, Patrizia Sabatelli, Rita Selvatici, Adele D’Amico, Marika Pane, Giulia Ricci, Gabriele Siciliano, Silvana Tedeschi, Antonella Pini, Liliana Vercelli, Domenico De Grandis, Eugenio Mercuri, Enrico Bertini, Luciano Merlini, Tiziana Mongini, Alessandra Ferlini

**Affiliations:** 1 Section of Medical Genetics, Department of Experimental and Diagnostic Medicine, University of Ferrara, Ferrara, Italy; 2 Istituto Di Genetica Molecolare-CNR, Istituto Ortopedico Rizzoli, Bologna, Italy; 3 Department of Laboratory Medicine, Unit of Molecular Medicine, Bambino Gesù Hospital, Rome, Italy; 4 Department of Child Neurology and Psychiatry, Catholic University, Rome, Italy; 5 Department of Neuroscience, Neurological Clinic, University of Pisa, Pisa, Italy; 6 Laboratory of Medical Genetics, I.R.C.C.S. Foundation Ca’ Granda, Maggiore Hospital, Policlinico, Milan, Italy; 7 Child Neurology and Psychiatry Unit, Maggiore Hospital, Bologna, Italy; 8 Neuromuscular Center, S.G. Battista Hospital, University of Turin, Turin, Italy; 9 Division of Neurology, Department of Neuroscience, Civile Hospital Santa Maria della Misericordia, Rovigo, Italy

**Keywords:** Dystrophinopathy, Female carriers, X-inactivation, Transcriptional balancing

## Abstract

**Background:**

Although Duchenne and Becker muscular dystrophies, X-linked recessive myopathies, predominantly affect males, a clinically significant proportion of females manifesting symptoms have also been reported. They represent an heterogeneous group characterized by variable degrees of muscle weakness and/or cardiac involvement. Though preferential inactivation of the normal X chromosome has long been considered the principal mechanism behind disease manifestation in these females, supporting evidence is controversial.

**Methods:**

Eighteen females showing a mosaic pattern of dystrophin expression on muscle biopsy were recruited and classified as symptomatic (7) or asymptomatic (11), based on the presence or absence of muscle weakness. The causative *DMD* gene mutations were identified in all cases, and the X-inactivation pattern was assessed in muscle DNA. Transcriptional analysis in muscles was performed in all females, and relative quantification of wild-type and mutated transcripts was also performed in 9 carriers. Dystrophin protein was quantified by immunoblotting in 2 females.

**Results:**

The study highlighted a lack of relationship between dystrophic phenotype and X-inactivation pattern in females; skewed X-inactivation was found in 2 out of 6 symptomatic carriers and in 5 out of 11 asymptomatic carriers. All females were characterized by biallelic transcription, but no association was found between X-inactivation pattern and allele transcriptional balancing. Either a prevalence of wild-type transcript or equal proportions of wild-type and mutated RNAs was observed in both symptomatic and asymptomatic females. Moreover, very similar levels of total and wild-type transcripts were identified in the two groups of carriers.

**Conclusions:**

This is the first study deeply exploring the *DMD* transcriptional behaviour in a cohort of female carriers. Notably, no relationship between X-inactivation pattern and transcriptional behaviour of *DMD* gene was observed, suggesting that the two mechanisms are regulated independently. Moreover, neither the total *DMD* transcript level, nor the relative proportion of the wild-type transcript do correlate with the symptomatic phenotype.

## Background

Duchenne and Becker muscular dystrophies (DMD, MIM#310200 and BMD, MIM#300376) are X-linked recessive conditions caused by mutations in the dystrophin gene. Both DMD and BMD usually affect males, with the majority of female carriers of *DMD* mutations being asymptomatic, often presenting with high serum creatine kinase (CK) levels as the only clinical sign. Nevertheless, a certain number of female carriers, defined as “manifesting” or “symptomatic”, do develop symptoms of the disease, which vary from a mild muscle weakness to a DMD-like clinical course; the muscle weakness is generally asymmetric and proximally distributed, and the age of onset is extremely variable [1,2]. Manifesting carriers may also present cardiac pathology (dilated cardiomyopathy), either alone or in addition to the muscle weakness [3,4]. The percentage of symptomatic carriers has been estimated in the range of 2.5-7.8% [1,2], based on skeletal muscle clinical involvement. In contrast, a study conducted in the Netherlands, taking into account not only muscle symptoms but also pure cardiac presentation, revealed a higher proportion of carriers manifesting symptoms, i.e. 22% [5].

Peculiar chromosomal aberrations are well known to be associated with clinical manifestations in DMD female carriers; for example Turner syndrome (45,X) associated with a *DMD* gene mutation on the single X chromosome [6], uniparental disomy of the X chromosome carrying the *DMD* mutation [7] and balanced X-autosome translocations with a breakpoint in the dystrophin gene.

X chromosome inactivation (XCI) represents the transcriptional silencing of the majority of genes on one of the X chromosomes in mammalian females. The choice of the X chromosome to be inactivated occurs in an early stage during embryonic development and the process is random and clonally inherited. Skewed X-inactivation is the preferential inactivation of one of the two X chromosomes; it may occur either by chance (primary non-random X-inactivation) or as a result of secondary cell selection [8,9]. In the case of carriers of X-autosome translocations, it is generally accepted that the manifestations of the disease result from a non-random X-inactivation pattern, with the derivative X remaining active as a saving mechanism against the monosomy of autosomal regions [10,11]. Different patterns of X-inactivation have also been demonstrated in pairs of clinically discordant monozygotic female twins heterozygous for dystrophin gene mutations [12-14]. Based on this evidence, skewed X-inactivation has classically been considered as the mechanism most likely to explain the dystrophic phenotype in female carriers, and this hypothesis has been lent weight by various X-inactivation studies performed on DNA from peripheral blood lymphocytes [15,16]. Nevertheless, other studies based on X-inactivation tests, performed on either peripheral blood [17-20] or muscle tissue [21], have highlighted the absence of a clear correlation between X-inactivation pattern and phenotype, reporting manifesting carriers with both skewed and completely random X-inactivation.

Hence, in order to explore the controversial issue of the pathogenic mechanism underlying clinical manifestation in DMD female carriers, we set out to compare the X-inactivation pattern with *DMD* allele transcription balancing in muscle tissue from 7 symptomatic and 11 asymptomatic carriers featuring fully characterized *DMD* gene mutations.

Surprisingly, this analysis revealed a complete lack of relationship between X-inactivation, transcription balancing and phenotype. In fact, the occurrence of skewed X-inactivation in muscle, inferred by studying androgen receptor (*AR*) gene methylation status, did not correlate with the symptomatic phenotype. In addition, transcriptional representation of the mutated *DMD* alleles in muscle failed to mirror the X-inactivation pattern, suggesting that these two mechanisms are independently regulated. In conclusion, neither skewed X-inactivation nor preferential expression of the mutated *DMD* allele enables prediction of a dystrophinopathic phenotype.

## Methods

### Sample

Eighteen females presenting a mosaic pattern of dystrophin expression under immunohistochemical analysis were enrolled in the study, which was approved by the local ethics committee (document n.9/2005).

Females were recruited in the course of regular NM clinic in different collaborating centers in the period between 2004 and 2010, and they underwent muscular biopsy due to persistent high CK or other signs and symptoms of the disease. All the analysed females were unrelated cases.

According to the literature, female carriers were classified as (1) symptomatic, when presenting with muscle weakness and/or dilated cardiomyopathy or (2) asymptomatic, when characterized by the presence of high CK levels and/or minor myopathic signs like muscle cramps and myalgia, but no muscle weakness or dilated cardiomyopathy [5,19].

### Mutation Analysis

Genomic DNA was extracted from peripheral blood by Biorobot Qiagen (Qiagen, Chatsworth, CA), after informed consent.

Mutation analysis was performed by Multiplex Ligation-dependent Probe Amplification (MLPA), to detect deletions and duplications, and direct sequencing, to identify point mutations. MLPA analysis was carried out with SALSA probemix 034 and 035 according to the manufacturer’s recommendations (MRC Holland, Amsterdam, Netherlands), thereby consenting the copy-number screening of all 79 dystrophin exons. PCR products were analysed on an ABI 3130 automated sequencer using Genescan software (Applied Biosystems). The dosage quotient was calculated as previously described [22].

For sequence analysis, dystrophin exons, including 3’ and 5’ intron boundaries, were PCR amplified. Amplification reactions were performed as previously described [23]. All PCR products were purified and sequenced on an ABI 3130 automated sequencer.

Comparative Genomic Hybridization (CGH) analysis by DMD-CGH microarray was performed as previously described [24].

### X-inactivation Studies

Genomic DNA was extracted from muscle tissue using the QIAblood Kit procedure (Qiagen, Chatsworth, CA). The (CAG)_n_ repeat at the *AR* locus was PCR-amplified in a reaction containing: 200 ng DNA, 10 mM Tris–HCl, 50 mM KCl, 1.5 mM MgCl_2_, 0.2 mM of each dNTP, 175 ng of each primer (forward: 5’-TCCAGAATCTGTTCCAGAGCGTG-3’; reverse: 5’-CTGTGAAGGTTGCTGTTCCTCAT-3’), 1.25U Taq polymerase (Invitrogen), under the following conditions: 94°C for 5 min; 28 cycles of 94°C for 45 sec, 60°C for 30 sec, 72°C for 30 sec. Amplification products were analyzed on an ABI 3130 automated sequencer and *AR* alleles were determined by Genemapper software (Applied Biosystems). The X-inactivation pattern was investigated in carriers informative for the (CAG)_n_ repeat. Analysis was performed in duplicates by semi-quantification of the relative inactivation of *AR* alleles [15]. 1 μg of DNA was digested with 10U HpaII and 10U CfoI (Boehringer Mannheim) for 2 h at 37°C. Enzymes were heat-inactivated and digested DNA samples were purified with Microcon columns; 200 ng were amplified as described above. PCR products from undigested and digested samples were run in parallel on ABI 3130 sequencer. The ratio between the two alleles of the undigested sample was calculated and the resulting correction factor was applied to allele values in the digested samples. The degree of X-inactivation was calculated by normalizing the sum of the two alleles in the digested sample to 100%. Results obtained from either peak height or peak area were compared and very similar values were obtained. X-inactivation pattern was considered as random for ratios ≤80:20, moderately skewed for ratios >80:20 and ≤90:10, and highly skewed for ratios >90:10, according to Amos-Landgraf et al., 2006 [25].

### RNA Analysis

Total RNA was isolated from skeletal muscle biopsy samples using the RNeasy Kit (Qiagen, Chatsworth, CA) according to the manufacturer’s instructions. Reverse transcription and cDNA amplification (RT-PCR) was performed as previously described [23]. The dystrophin transcript was analysed using appropriate pairs of oligonucleotides (sequences are available upon request). All PCR fragments were purified and sequenced.

In order to assess the proportion of wild-type vs. deleted/duplicated transcripts, a relative quantification was performed using an Agilent High Sensitivity DNA chip (Agilent Technologies, Santa Clara, CA), which allows the sizing and quantification of cDNA samples in the 5–500 pg/μL concentration range. Different numbers of amplification cycles were tested: 25 cycles reactions were performed in order to avoid quantification during the plateau phase of PCR. Amplification products were analysed by an Agilent 2100 Bioanalyzer. The relative percentages were calculated as the ratio between each type of transcript (wild-type or mutated) and the sum of all transcripts (wild-type + mutated).

A relative quantification of dystrophin transcript was performed by real-time PCR. Taqman probes specific for dystrophin exon 12 and exon 55 and a commercially available Taqman expression assay for beta actin (ACTB Endogenous Control) (Applied Biosystems) were used. Samples were analyzed in triplicate on the Applied Biosystems Prism 7900 system. The relative quantification of the target sequences in respect to beta actin was performed by the comparative CT method (ΔΔCT Method) (Applied Biosystems User Bullettin #2); cDNAs from two control females were used as calibrators. Each experiment was performed in duplicate.

### *SPP1* Polymorphism Genotyping

17 out of 18 carriers were genotyped for the *SPP1* (osteopontin) intragenic variant *T/G* in the promoter region (dbSNP: rs28357094). The polymerase chain reaction was performed on genomic DNA using a specific pair of primers (forward: 5’-AAGTGCTCTTCCTGGATGCTG-3’; reverse: 5’-CTCCTGCTGCTGCTGACAAC-3’). All PCRs were run in a final volume of 25 μl, containing 100 ng genomic DNA, 20 mM Tris–HCl (pH 8.4), 50 mM KCl, 1.5 mmol/l MgCl_2_, 0.2 mmol/l dNTPs, 5U Taq DNA Polymerase (Invitrogen) and 0.4 μl of each primer.

### SDS–PAGE Electrophoresis and Immunoblotting Analysis

20-μm thick frozen muscle sections from each patient and a control female were homogenized for 2 hours at 4°C with a RIPA lysis buffer containing: 50 mM Tris/HCl, 150 mM NaCl, 1 mM EDTA, 1% NP40, 0.1% SDS, 0.5% deoxycholic acid, and an EDTA-free protease inhibitor cocktail tablet (Roche), and then centrifuged at 1500 g for 10 min. Protein content was determined by the bicinchoninic acid method (Bicinchoninic protein assay, Pierce) [26]. Equal amounts of protein (30 μg) were subjected to gel electrophoresis (150 V for 1 h) on a 6% gel, and then electrophoretically transferred to a nitrocellulose membrane (100 V for 2 h). After transfer, the gel was stained with Coomassie brilliant blue solution.

Membrane was blocked with non-fat dried milk for 1 h at room temperature and incubated overnight at 4°C with the specific antibodies DYS2 (a mouse monoclonal antibody to the carboxy terminal region of dystrophin, 1:200, NovoCastra, Newcastle, U.K.) and DYS1 (a mouse monoclonal antibody to the rod domain of dystrophin, 1:500, NovoCastra, Newcastle, U.K.). After washing, the membrane was incubated with horseradish peroxidase-conjugated goat anti-mouse 1:40000, for 1 h at room temperature. Immunocomplexes were visualized by means of the ECL Advance Western Blotting Detection Kit (Amersham Pharmacia Biotech, Buckinghamshire, UK); exposure time on X-ray film was from less than 10 seconds to 1 minute. Densitometric analysis of autoradiographic bands was performed with a Bio-Rad densitometer GS700 and expressed as absorbance (A).

## Results

Eighteen females presenting a mosaic pattern of positive and negative dystrophin fibres on muscle biopsy were identified. These subjects were allocated to one of two phenotype groups: (1) symptomatic and (2) asymptomatic, based on criteria reported in the Methods section.

Clinical data are summarized in Table 1. Among the 11 asymptomatic females, high serum CK levels were detected in all cases, either alone or in association with other minor myopathic signs like calf hypertrophy (in 5 females) or myalgia (in 1 female). Raised serum CK levels were also detected to a significantly similar extent in the 7 symptomatic females.

**Table 1:** Clinical and biochemical data of symptomatic and asymptomatic female carriers

**Carrier ID**	**Age at examination**	**Classification**	**Age at onset**	**Muscle weakness**	**Other signs**	**CK levels (U/l)**	**Echocardiogram**	**Muscle biopsy**		
								**Age at biopsy**	**Muscle**	**Muscle pathology**
**C1**	5y8m	Symptomatic	2y	Yes: progressive, proximal, symmetric.	hCK No running, no jumping, positive Gowers sign	13430 at 6y	Normal	6y	Quadriceps	Moderate increase of endomysial fibrous tissue, moderate number of necrotic fibers, mild increase of internal nuclei, moderate number of hypercontracted fibers.
**C2**	34y	Symptomatic	na	Yes	hCK	1050 at 34y	Normal	34y	Tibialis anterior	Marked fiber size variation, moderate increase of endomysial fibrous tissue, increase of internal nuclei.
**C3**	4y	Symptomatic	4y	Yes: non progressive, proximal, symmetric. Main districts: limbs, trunk.	hCK Myalgia	2200 at 4y3m	nd	4y	Quadriceps	Rare internal nuclei.
**C4**	25y	Symptomatic	23y	Yes: progressive, proximal, asymmetric. Main districts: limbs, girdles.	hCK Calves hypertrophy Positive Gowers sign	3034 at 25y	nd	25y	Quadriceps	Marked fiber size variation, marked increase of endomysial fibrous tissue, some necrotic fibers.
**C5**	43y	Symptomatic	43y	Yes: progressive, proximal, symmetric. Main districts: limb girdles and distal muscles of lower limbs.	hCK	933 at 43y	nd	43y	Left deltoid	Moderate increase of endomysial fibrous tissue, marked fiber size variation, numerous internal nuclei.
**C6**	27y	Symptomatic	11y	Yes: progressive, proximal, symmetric. Main districts: girdles	hCK Anserine walking	714 at 27y	nd	27y	Quadriceps	Marked fiber size variation, increase of endomysial fibrous tissue, several internal nuclei, some necrotic fibers.
**C7**	9y	Symptomatic	na	Yes: symmetric, diffuse.	hCK Calves hypertrophy	6500 at 2y	nd	9y	Quadriceps	Moderate fiber size variation, several internal nuclei.
**C8**	20y	Asymptomatic	-	No	hCK Calves hypertrophy	>4000 at 7y	Normal	7y	Quadriceps	Moderate fiber size variation, hypotrophic fibers and degenerating-regenerating fibers.
**C9**	34y	Asymptomatic	-	No	hCK Mild calves hypertrophy	1654 at 16y	Normal	9y	Quadriceps	Mild myopathic signs.
**C10**	35y	Asymptomatic	-	No	hCK (at 32y and 35y)	Not reported	nd	35y	Quadriceps	Moderate fiber size variation.
**C11**	47y	Asymptomatic	-	No	hCK	899 at 47y	nd	47y	Quadriceps	Fiber size variation with hypertrophic fibers, internal nuclei.
**C12**	24y	Asymptomatic	-	No	hCK Calves hypertrophy	1295 at 24y	Normal	24y	Quadriceps	Very mild fiber size variation, some internal nuclei.
**C13**	21y	Asymptomatic	-	No	hCK Calves hypertrophy	10800 at 21y	Left ventricle at the upper limit	21y	Quadriceps	Moderate fiber size variation, increase of endomysial fibrous tissue, numerous internal nuclei, necrotic fibers.
**C14**	6y	Asymptomatic	-	No	hCK Myalgia	4439 at 6y	Normal	6y	Quadriceps	Fiber type variation, central nuclei, absence of inflammation.
**C15**	13y	Asymptomatic	-	No	hCK Learning difficulties	1732 at 13y	nd	13y	Quadriceps	Mild fiber size variation, increase in internal nuclei.
**C16**	17y	Asymptomatic	-	No	hCK Mild calves hypertrophy	2700 at 17y	nd	3y	Tibialis anterior	Dystrophic pattern.
**C17**	5y	Asymptomatic	-	No	hCK	8207 at 2y	Normal	2y	Quadriceps	Fiber size variation, mild increase of endomysial fibrous tissue, some groups of regenerating fibers.
**C18**	6y	Asymptomatic	-	No	hCK	10242 at 6y	Normal	6y	Quadriceps	Moderate increase of endomysial fibrous tissue, fiber size variation, several internal nuclei, some degenerating fibers.

*hCK*: high CK; *na*: not available; *nd*: not determined.

In this latter group, the age at symptom onset varied from 2 to 43 years. Muscle weakness was predominantly proximal and symmetric. Echocardiographic examination was performed in 9 females (2 symptomatic, 7 asymptomatic), yielding results within normal limits in all cases.

**Figure 1:**
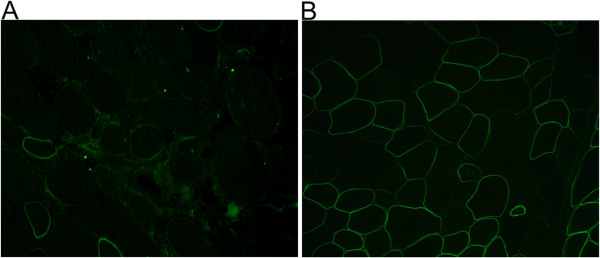
**Immunohistochemical staining of muscle sections from carriers 1 and 2. A**) Immunolabeling in symptomatic carrier 1 with NCL-DYS3 antibody (NH_2_-terminal) shows absence of staining in the majority of muscle fibers (approximately 80% in the whole section). **B**) Immunolabeling in symptomatic carrier 2 with DYS2 antibody (COOH-terminal) shows a mosaic pattern of dystrophin expression with coexistence of positive fibers, fibers with reduced or discontinuous staining and fibers with absence of labeling.

One or more histopathological changes were observed in all muscle biopsy samples: the most common findings included variation in fibre size, increase in endomysial fibrous tissue, increased number of central nuclei, and presence of necrosis and regeneration. Immunohistochemical staining showed a mosaic pattern with populations of dystrophin-positive fibres, fibres with reduced or discontinuous staining, and dystrophin-negative fibres (Figure 1). Positive fibres count on multiple biopsy sections was not possible due to the limited amount of muscle tissue available.

### *DMD *Mutations in Female Carriers

**Figure 2:**
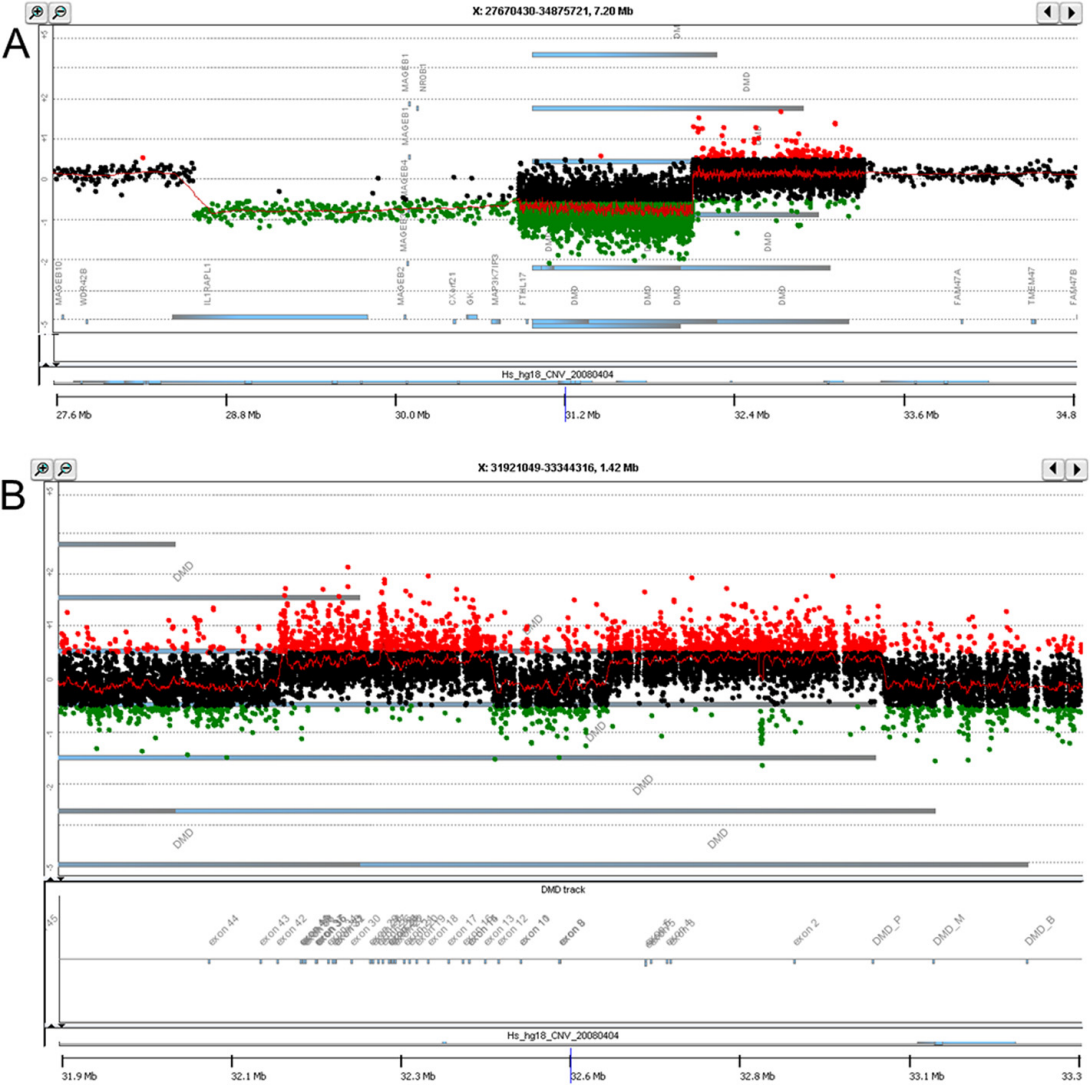
**DMD-CGH array profile in carriers 16 and 8. A**) In carrier 16, a deletion of 3.5 Mb was detected (chrX:g.(28.671.682_28.671.742)_(32.170.481_32.170.541)del). The 5’ breakpoint is located within intron 43 of the *DMD* gene and deletion covers the following downstream genes: *FTHL17, MAP3K7IP3, GK, CXorf21, NR0B1, MAGEB4, MAGEB3, MAGEB2, MAGEB1, IL1RAPL1. ***B**) In carrier 8, CGH analysis confirmed a non-contiguous duplication involving exons 1P-7 (chrX:g.(33.068.711_33.068.771)_(32.684.693_32.684.750)dup) and exons 13–42 (chrX:g.(32.523.766_32.523.826)_(32.228.415_32.228.475)dup). A polymorphic CNV is also visible within intron 2.

Genomic analysis consented detection of the causative *DMD* mutation in all subjects analysed (Table 2). 10 deletions and 5 duplications of one or more exons were identified by MLPA. Most deletions were clustered in the distal hot-spot region of the gene; 8 were out-of-frame and 2 extended beyond exon 79 (carriers 15 and 16). For female 16, carrying an exon-44-to-79 deletion, CGH-array allowed us to determine the 3’ breakpoint, and a large 3.5 Mb deletion, also including *IL1RAPL-1* gene, was identified (Figure 2a).

**Table 2:** Molecular characterization, X-inactivation pattern and transcriptional analysis results in symptomatic and asymptomatic carriers

**Carrier ID**	**Classification**	**DMD mutation**^**(a)**^	**X-inactivation pattern**	**RNA profiling**	**Transcripts representation (wt:mutated)**	**Total transcript level**	**WT transcript level**
**C1**	Symptomatic	t(X;9)(p21.1;p22.1)	100:0 (blood) 100:0 (sk.m.)	Biallelic transcript (biallelic pol. 3’-UTR)	nd	nd	nd
**C2**	Symptomatic	Dup exons 3–7 c.94-?_649 + ?dup (out of frame)	60:40	WT + DUP 3–7 transcripts	nd	nd	nd
**C3**	Symptomatic	Dup exons 5–7 c.265-?_649 + ?dup (out of frame)	75:25	WT + DUP 5–7 transcripts	70:30	nd	nd
**C4**	Symptomatic	Del exons 8–9 c.650-?_960 + ?del (out of frame)	86:14	WT + DEL 8–9 transcripts	50:50	17% (ex 12) 12% (ex 55)	9% (ex 12) 6% (ex 55)
**C5**	Symptomatic	Del exon 44 c.6291-?_6438 + ?del (out of frame)	Non Informative	WT + DEL 44 transcripts	88:12	109% (ex 12) 31% (ex 55)	96% (ex 12) 27% (ex 55)
**C6**	Symptomatic	Del exon 54 c.7873-?_8027 + ?del (out of frame)	54:46	WT + DEL 54 transcripts	89:11	46% (ex 12) 78% (ex 55)	41% (ex 12) 69% (ex 55)
**C7**	Symptomatic	Del exons 8–48 c.650-?_7098 + ?del (out of frame)	34:66	WT + DEL 8–48 transcripts	nd	nd	nd
**C8**	Asymptomatic	Dup 1P-7 and dup 13–42 chrX:g. (33.068.711_33.068.771) _(32.684.693_32.684.750)dup; g.(32.523.766_32.523.826)_(32.228.415_32.228.475)dup	73:27 (blood) 97:3 (sk.m.)	Biallelic transcript (biallelic ex 21 SNP)	nd	nd	nd
**C9**	Asymptomatic	Dup exon 2 c.32-?_93 + ?dup (out of frame)	86:14	WT + DUP 2 transcripts	74:26	36% (ex 12) 36% (ex 55)	27% (ex 12) 27% (ex 55)
**C10**	Asymptomatic	Dup exons 3–44 c.94-?_6438 + ?dup (in frame)	85:15	DUP 3–44 transcript (junction PCR)	nd	nd	nd
**C11**	Asymptomatic	Del exons 48–52 c.6913-?_7660 + ?del (out of frame)	23:77	WT + DEL 48–52 transcripts	52:48	33% (ex 12) 48% (ex 55)	17% (ex 12) 25% (ex 55)
**C12**	Asymptomatic	Del exons 46–51 c.6615-?_7542 + ?del (out of frame)	65:35	WT + DEL 46–51 transcripts	49:51	118% (ex 12) 128% (ex 55)	58% (ex 12) 63% (ex 55)
**C13**	Asymptomatic	Del exons 49–50 c.7099-?_7309 + ?del (out of frame)	44:56	WT + DEL 49–50 transcripts	87:13	43% (ex 12) 34% (ex 55)	38% (ex 12) 29% (ex 55)
**C14**	Asymptomatic	Del exon 52 c.7543-?_7660 + ?del (out of frame)	87:13	WT + DEL 52 transcripts	65:35	48% (ex 12) 92% (ex 55)	31% (ex 12) 60% (ex 55)
**C15**	Asymptomatic	Del exons 50–79 c.7201-?_(*2691_?)del	81:19	Biallelic transcript (biallelic ex 37 SNP)	nd	nd	nd
**C16**	Asymptomatic	Del exons 44–79 chrX:g.(28.671.682_28.671.742)_(32.170.481_32.170.541)del	53:47	Biallelic transcript (biallelic ex 21 SNP; monoallelic ex 37 SNP)	nd	nd	nd
**C17**	Asymptomatic	c.1615C > T (ex 14) p.R539X	42:58	WT + 1615C > T transcripts	nd	nd	nd
**C18**	Asymptomatic	c.10033C > T (ex 69) p.R3345X	70:30	WT + 10033C > T transcripts	nd	nd	nd

*Sk.m*.: skeletal muscle; *WT*: wild-type; *MUT*: mutated; *nd*: not determined.

^a^ The DNA mutation numbering is based on cDNA sequence with a “c.” symbol before the number with +1 corresponding to the A of the ATG translation initiation codon in the respective reference sequence (GenBank:M18533); in carriers C8 and C16 nucleotides are numbered according to March 2006 Human Reference Sequence, NCBI Build 36.1, hg18.

Duplications were primarily located at the 5’-region of the gene; 3 were out-of-frame and 1 represented by a large in-frame duplication spreading from exon 3 to 44. A non-contiguous duplication of exons 1P-7 and exons 13–42 was also identified by MLPA and confirmed by CGH-array analysis (Figure 2b).

In two females negative for deletions and duplications, nonsense mutations, occurring at exons 14 and 69, were identified by sequencing.

The remaining female (carrier 1) presented a balanced translocation involving chromosome Xp21.1 and chromosome 9; DMD-CGH-array showed normal dosage of the *DMD* genomic locus (data not shown).

### X-Inactivation Pattern in Skeletal Muscle

17 out of the 18 female carriers were found to be heterozygous at the *AR* locus, and the X-inactivation pattern was assessed (Table 2); two independent experiments were performed with reproducible results. 4 symptomatic females showed clearly unbiased X-inactivation (carriers 2, 3, 6, and 7), 1 featured a moderately skewed pattern (carrier 4), and 1 highly skewed X-inactivation (carrier 1); among asymptomatic females, 6 showed random X-inactivation (carriers 11, 12, 13, 16, 17, and 18), 4 a moderately skewed pattern (carriers 9, 10, 14, and 15), and 1 highly skewed X-inactivation (carrier 8). The pattern obtained in the muscle DNA of the two highly skewed samples (carriers 1 and 8) was compared with that observed in their blood DNA. In carrier 1, bearing the X;9 translocation, a total skewing of 100:0 was confirmed in blood, while the pattern observed in the muscle of carrier 8 contrasted with the random inactivation observed in lymphocytes.

### Transcriptional Behaviour of* DMD* Alleles

Transcriptional analysis was performed on muscle biopsy samples from all subjects (Table 2).

All females, irrespective of their clinical status, showed the same transcriptional behaviour, with the co-existence of both wild-type and mutated transcripts.

The presence of biallelic transcripts was demonstrated by RT-PCR in all subjects carrying deletions, with the exception of females 15 and 16, who carry deletions extending into the 3’-UTR. In these cases, the expression of common polymorphisms of the *DMD* gene, found to be heterozygous at genomic level, was evaluated: in carrier 15 (del 50–79) the biallelic expression of *c.5234G>A* polymorphism in exon 37 demonstrated the presence of a biallelic transcript; carrier 16 (del 44–79) manifested biallelic expression of *c.2645G>A* polymorphism in exon 21 and monoallelic expression of *c.5234G>A* polymorphism in exon 37. This unexpected transcriptional behaviour could suggest the occurrence of splicing with an unknown gene downstream of the deletion breakpoint and the consequent generation of a chimeric transcript.

The presence of both wild-type and mutated transcripts was assessed by RT-PCR in carriers presenting duplications, with the exception of females 8 and 10; amplification of the entire duplicated region was not possible in carrier 10, but the presence of the mutated transcript was however demonstrated by amplification of the duplication junction (ex44-ex3). Due to the absence of heterozygous exonic polymorphisms, we were only able to infer the presence of the wild-type transcript. In the subject possessing a non-contiguous duplication (carrier 8), the presence of a biallelic transcript was demonstrated by the biallelic expression of *c.2645G>A* polymorphism in exon 21.

RNA studies in females 17 and 18, both carrying nonsense mutations, showed the presence of both wild-type and mutated transcripts.

In carrier 1, the presence of a biallelic transcript, comprising one originating from the normal X-chromosome and one from the derivative X-chromosome, was demonstrated by the biallelic expression of a rare polymorphism in the 3’-UTR of the gene (*c.11058+22 del13nt*). Since this variant was the only polymorphism found to be heterozygous on genomic DNA, we could not rule out the possibility that the observed biallelism reflects the transcription of a 3’ isoform of dystrophin, rather than the full-length RNA.

Semi-quantitative analysis (Table 2) revealed a prevalence of the wild-type transcript in 3 symptomatic (carriers 3, 5 and 6) and 3 asymptomatic (carriers 9, 13, and 14) females and equal proportions of wild-type and mutated mRNAs in 1 symptomatic (carrier 4) and 2 asymptomatic (carriers 11 and 12) females.

**Figure 3:**
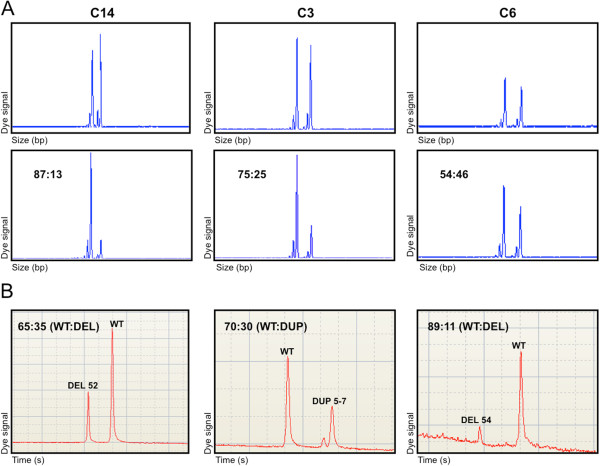
**Comparison between X-inactivation pattern and transcript quantification. A**) X-inactivation assay based on *AR* gene methylation. The upper line corresponds to PCR products of undigested muscle DNA, showing the two alleles at *AR* locus; the lower shows the products of DNA amplification after digestion with the methylation-sensitive enzymes HpaII and CfoI. **B**) RT-PCR analysis with a High Sensitivity DNA chip (Agilent). The ratios between wild-type and mutated transcripts are indicated. Carrier 14 is an asymptomatic female with a moderately skewed X-inactivation pattern of 87:13 and a transcript ratio of 65:35 (wt:deleted); symptomatic carrier 3 presented X-inactivation of 75:25 and a transcript ratio of 70:30 (wt:duplicated); carrier 6 is a symptomatic female featuring random X-inactivation of 54:46 and transcript ratio of 89:11 (wt:deleted).

**Figure 4:**
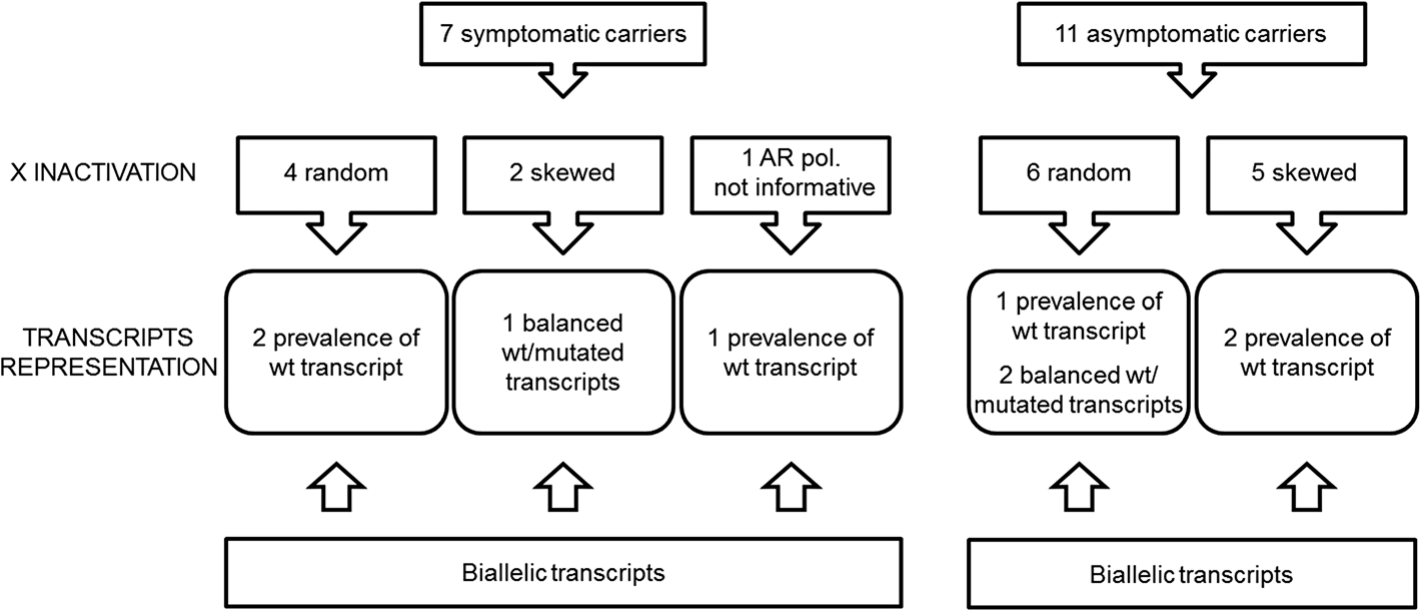
**Schematic representation of results from X-inactivation and transcriptional analysis.** This schematic summarizes the results of X-inactivation studies and transcript quantification in the two groups of symptomatic and asymptomatic carriers, and highlights the absence of a relationship between X-inactivation pattern, transcriptional behaviour and dystrophic phenotype in female carriers.

In the majority of cases, no relationship was found between the X-inactivation pattern and the transcriptional level of dystrophin alleles (Figure 3 and Figure 4).

**Figure 5:**
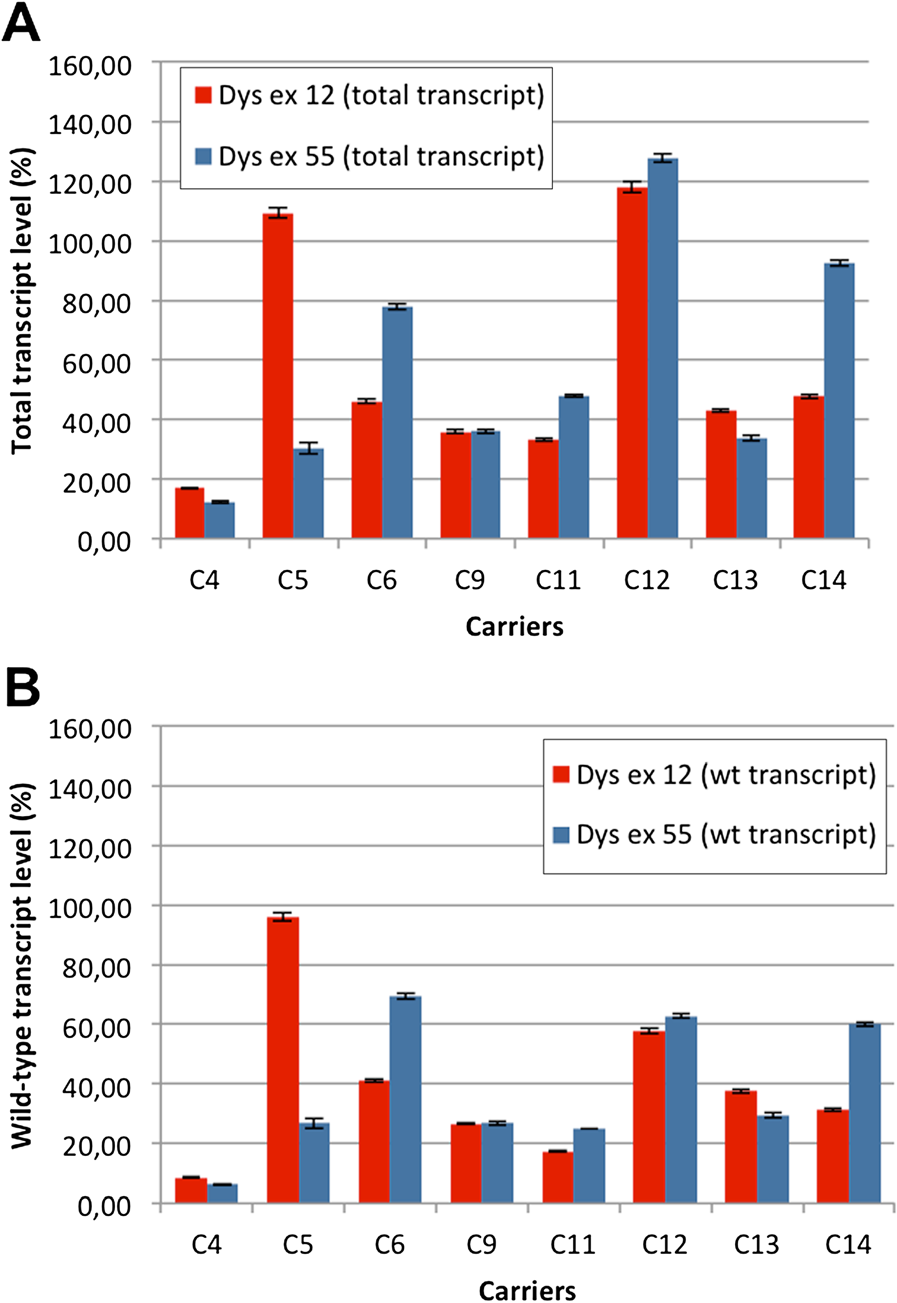
**Relative quantification of dystrophin transcript by real-time PCR.A**) Histograms represent total transcript level of dystrophin gene in 8 female carriers, calculated on dystrophin exon 12 and exon 55, in respect to two control females (their mean value referred as 100%). Error bars represent mean ± SD. **B**) Transcriptional level of wild-type dystrophin allele, calculated from the relative ratio of wild-type and mutated transcripts.

Eight carriers were further investigated by real-time PCR in order to quantify the level of dystrophin transcription, in respect to control females. Total transcript quantification was performed by two different real-time assays, on dystrophin exon 12 and exon 55 (Figure 5a). The selected exons are not implicated in deletion or duplication in any of the carriers analyzed; the level of wild-type transcript was derived from the relative ratio of wild-type and mutated transcripts previously calculated (Figure 5b). No relationship was identified between phenotype and dystrophin transcription level. Among symptomatic carriers, the percentage of wild-type transcript ranged between 9% and 96% (exon 12 assay) and between 6% and 69% (exon 55 assay), in respect to control females; similar levels were observed among asymptomatic carriers, with wild-type transcript levels comprised between 17% and 58% (exon 12 assay) and between 25% and 63% (exon 55 assay), in respect to controls.

### *SPP1* Polymorphism Genotyping

Genotype analysis of the *SPP1* variant was performed in 17 subjects; *T* allele homozygosity was revealed in 5 out of 6 symptomatic carriers and *T* allele heterozygosity was detected in only one. In the asymptomatic group, *T* allele homozygosity was detected in 7 out of 11 subjects and heterozygosity in 3 out of 11; the presence of an homozygous *G* allele was found only in one asymptomatic carrier.

### Western Blot Results

**Figure 6:**
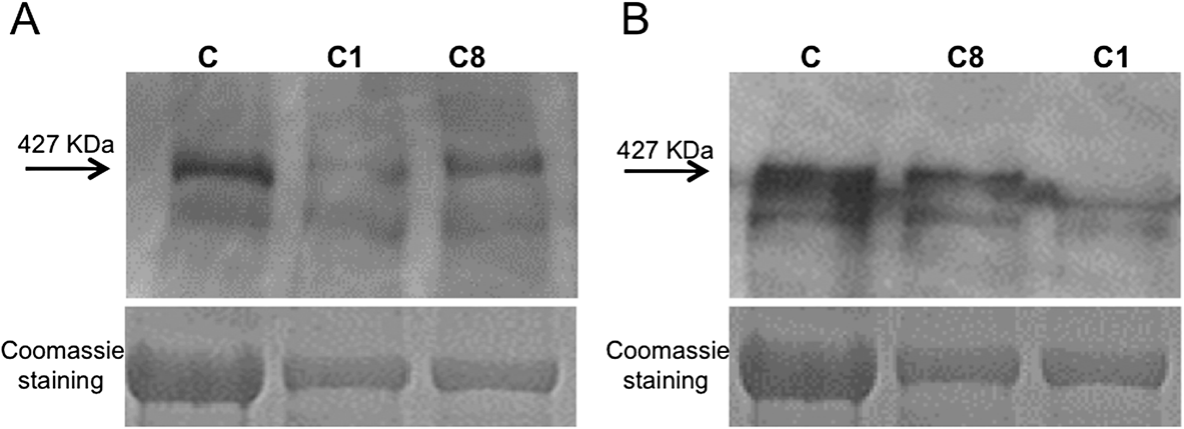
**Immunoblotting in carriers 1 and 8 and Coomassie staining for protein quantification by densitometric analysis. A**) Immunoblot with DYS2 antibody (directed against carboxy terminal region of dystrophin) shows protein levels of 15% for carrier 1 and 70% for carrier 8, with respect to control. **B**) Immunoblot with DYS1 antibody (directed against rod domain) reveals 11% and 87% of normal protein levels for carriers 1 and 8, respectively.

For carriers 1 and 8, both presenting skewed X-inactivation but different phenotypes, we performed a precise quantification of dystrophin protein content in quadriceps muscles via immunoblot using DYS2 and DYS1 antibodies. This revealed the presence of a protein of the correct size in both patient samples, but relative quantification by densitometric analysis demonstrated that muscles from subjects 1 (manifesting) and 8 (asymptomatic) contained, respectively, 15% and 70% of normal, as compared to the control sample (Figure 6a). These proportions were confirmed with DYS1 antibody, which revealed 11% and 87% of normal protein levels for carriers 1 and 8, respectively (Figure 6b).

## Discussion

In order to shed light on the mechanism responsible for the clinical manifestations in symptomatic female DMD carriers, we recruited a cohort of 18 female carriers of a mutation in the dystrophin gene, 7 symptomatic and 11 asymptomatic, and compared their genomic and transcription profiles.

In both symptomatic and asymptomatic females, immunostaining of muscle biopsy tissue with anti-dystrophin antibodies highlighted a mosaic pattern of dystrophin-positive and dystrophin-negative fibres, in addition to fibres with reduced dystrophin labelling. Results reported by other groups showed no correlation between the percentage of negative fibres and clinical phenotype [17,27-29].

Skewed X-chromosome inactivation has been proposed as a possible explanation for the presence of symptoms in manifesting carriers; based on this hypothesis, the X chromosome carrying the mutated dystrophin gene should be active in the majority of nuclei in muscles from symptomatic carriers.

X chromosome inactivation can be tested by different methods. Methylation analysis at the androgen receptor locus represents the most used assay: in contrast to direct methods based on expression analysis of polymorphic X-linked genes, *AR* assay is an indirect method based on differential methylation on active and inactive X chromosome; however it is considered an accurate test as high correlation has been demonstrated with results obtained from direct expression analysis. Despite the fact that several studies assessing the role of X-inactivation have been performed, their findings have been inconclusive and even contradictory. This disparity in results is partly due to the non-homogeneity of the criteria adopted in different studies, such as the use of different cut-off levels to define skewed X-inactivation [15,19,21,25]. Furthermore, these studies have frequently employed lymphocytes in evaluating X-inactivation, but these cells fail to mirror dystrophin expression, and considerable differences in X-inactivation pattern between different tissues from the same individual have been reported [21].

Nonetheless, the role of the X-inactivation pattern has been demonstrated in females with X-autosome translocation; cells featuring inactivation of the derivative X chromosome become partially monosomic for the translocated autosome, thereby conferring a selective advantage on cells having the derivative X chromosome active. This is the situation in our carrier 1, who features a balanced X;9 translocation; in this case the totally skewed pattern of 100:0 observed in her blood and muscle is not unexpected, and is in accordance with her severe phenotype. However, the absence of dystrophin labelling in the majority of fibres (80%), but not all as would be expected from a completely silenced normal X chromosome, raises the question of how and where dystrophin-positive fibres arise. Among the other symptomatic females informative for the polymorphism at the *AR* locus, the majority (4 out of 5) presented a random X-inactivation pattern, revealing a lack of relationship between X-inactivation pattern and clinical phenotype.

This is not surprising, as the absence of relationship between X chromosome inactivation pattern and phenotype has been highlighted for other X-linked disorders: in hemophilia A and B [30], Fabry disease [31] and myotubular myopathy [32] the occurrence of disease manifestations in females do not correlate with skewed X-inactivation.

Genomic analysis was integrated with RNA studies performed on muscle biopsy and revealed homogeneous transcriptional behaviour; in all carriers, both symptomatic and asymptomatic, the transcription of both mutated and wild-type mRNAs was observed. The coexistence of the two types of transcripts was demonstrated in almost all cases, either directly by RT-PCR or indirectly by the observation of the biallelic transcription of polymorphism-containing exons. Unfortunately, the huge size of the duplication and the absence of heterozygous exonic polymorphisms precluded identification of this evidence in carrier 10. Nonetheless, biallelic transcription was demonstrated in carriers 15 and 16, despite the presence of large deletions extending into the 3’-UTR of the gene, which might have reduced mRNA stability. In particular, we had the opportunity of further exploring carrier 16 by CGH-array; the 3’ breakpoint of the dystrophin rearrangement was thereby defined, and a 3.5 Mb deletion, extending outside the *DMD* genomic locus, was identified. In this case, the transcriptional behaviour seems to be very complex, presumably involving the formation of a fusion transcript comprising the *DMD* locus and another unidentified adjacent gene. However the evidence of monoallelic transcription of *c.5234G>A* polymorphism in dystrophin exon 37 suggests that the fusion breakpoint on the *DMD* gene transcript does not correspond to the breakpoint of the genomic deletion, which is located in intron 43.

The data gathered in the present study did highlight the absence of any relationship between X-inactivation pattern, transcriptional behaviour and dystrophic phenotype in females. Relative quantification of wild-type and mutated transcripts was performed in 9 carriers, revealing no relationship with the X-inactivation pattern. Among symptomatic females, this was particularly evident in carrier 4, characterized by skewed X-inactivation and similar levels of the two transcripts, and carrier 6, who, in contrast, was shown to possess a random X-inactivation pattern but a strong prevalence of the wild-type transcript. The absence of a relationship between X-inactivation pattern and transcription was also evident in asymptomatic carriers 11, 13, and 14.

The lack of relationship between phenotype and transcriptional behaviour is also evident from our results; among the 4 symptomatic carriers analysed with the High Sensitivity Chip, 3 showed the prevalence of the wild-type transcript over the mutated transcript, and 1 was characterized by similar levels of wild-type and mutated mRNAs. This discrepancy between transcript biallelic representation and X-inactivation strongly suggests that other, probably genetic, determinants may independently influence dystrophin transcription and X chromosome inactivation.

A genetic modifier of DMD severity in males has been recently described [33]; the *G* allele of the rs28357094 polymorphism in the osteopontin ( *SPP1*) promoter was demonstrated to be associated with increased muscle weakness and precocious loss of ambulation. Hence, we analysed this variant in our series of carriers. Despite the small size of the cohort precluding statistical significance, we did find similar proportions of *T* and *G* alleles in symptomatic ( *T* allele: 11/12; *G* allele; 1/12) and asymptomatic ( *T* allele: 17/22; *G* allele: 5/22) subjects.

Relative quantification of wild-type and mutated alleles was performed in females carrying out-of-frame mutations; these results could therefore be biased by nonsense-mediated decay of the mutated allele. In order to overcome this problem, we performed a relative quantification of wild-type transcript in respect to control females. Our data highlights the absence of relationship between phenotype and dystrophin transcriptional level. With the exception of carrier 4, characterized by very low dystrophin transcription rate (6-9% in respect to controls), in the other cases the levels quantified are very similar in symptomatic and asymptomatic carriers.

A reduction in dystrophin protein abundance appears to be a common feature in carriers: reduced protein levels have been described both in symptomatic [17,28] and asymptomatic females [21]. In our cohort of carriers, the manifesting female 1 for whom a precise protein quantification was performed showed a protein level of less than 15% of normal abundance; quantification in asymptomatic carrier 8 showed protein levels more than 70% of normal. These data raise the question of a possible relationship between dystrophin protein level and phenotype. This issue will need to be addressed in future studies of protein quantification with larger number of carriers.

## Conclusions

Considering our results along with those reported in the literature, X-inactivation aetiology has been definitively ruled out as an explanation of symptomatic phenotype in female carriers. For the first time we have shown that neither the transcriptional behaviour of *DMD* gene enables prediction of a dystrophinopathic phenotype: no relationship between phenotype and total *DMD* transcript level, or between phenotype and relative proportion of the wild-type transcript was observed in females. Interestingly, our study also shows the absence of a relationship between X-inactivation and transcriptional pattern of dystrophin, suggesting that *DMD* gene escape, to some extent, X chromosome inactivation. Our data suggest that the key role in determining a symptomatic phenotype in DMD female carriers may be played by the protein amount, fact that deserves further fine quantitative assays on larger patients cohort and relative muscle samples.

### Limitation section

#### Early age at clinical assessment

Three of the children in the asymptomatic group of females (C14, C17 and C18) were examined at a young age (6 years or younger), so that it is not possible to completely exclude that a phenotype will compare at later ages*.* A comparison between age-matched groups of carriers (symptomatic vs asymptomatic) would be desirable. Despite this limitation, even if we avoid to consider C14 (the unique young girl studied for RNA behaviour) the general conclusions of our analysis remain unchanged.

#### Lack of extensive protein data

We could provide Western blot data only for two females: one symptomatic (C1) and one asymptomatic (C8) carrier. No residual biopsy was available for females other than C1 and C8, after immunohistochemical analysis. The lack of extensive protein data represents a limitation of the present work. Nevertheless, the existence of a relationship between phenotype and dystrophin protein levels (emerging from WB results in females C1 and C8) is largely supported by evidence in the literature.

## Competing interests

The authors declare that they have no competing interests.

## Authors’ contributions

SB performed most of the experimental work (MLPA, RT-PCRs, inactivation studies, allele quantification on Agilent chip, real-time PCRs) and wrote the draft of the manuscript. FG supervised the work, performed part of the experimental work (RNA work and inactivation studies), critically revised the manuscript. CS performed *SPP1* SNP analysis. AA collected clinical information of enrolled patients. MB performed CGH studies. MSF performed Western Blot analysis. PS performed immunohistochemical analysis. RS performed sequencing analysis. AD, MP, GR, GS, ST, AP, LV, DD, EM, EB, LM, TM referred patients to our laboratory and provided clinical data. AF conceived the work and approved the final version of the manuscript. All authors read and approved the final manuscript.

## Pre-publication history

The pre-publication history for this paper can be accessed here:

http://www.biomedcentral.com/1471-2350/13/73/prepub
